# The Ansamycin Antibiotic, Rifamycin SV, Inhibits BCL6 Transcriptional Repression and Forms a Complex with the BCL6-BTB/POZ Domain

**DOI:** 10.1371/journal.pone.0090889

**Published:** 2014-03-04

**Authors:** Sian E. Evans, Benjamin T. Goult, Louise Fairall, Andrew G. Jamieson, Paul Ko Ferrigno, Robert Ford, John W. R. Schwabe, Simon D. Wagner

**Affiliations:** 1 Department of Biochemistry, University of Leicester, Leicester, United Kingdom; 2 Department of Cancer Studies and Molecular Medicine and MRC Toxicology Unit, University of Leicester, Leicester, United Kingdom; 3 Department of Chemistry, University of Leicester, Leicester, United Kingdom; 4 Section of Experimental Therapeutics, Leeds Institute of Molecular Medicine, University of Leeds, Leeds, United Kingdom; Institut de Recherches Cliniques de Montréal (IRCM), Canada

## Abstract

BCL6 is a transcriptional repressor that is over-expressed due to chromosomal translocations, or other abnormalities, in ∼40% of diffuse large B-cell lymphoma. BCL6 interacts with co-repressor, SMRT, and this is essential for its role in lymphomas. Peptide or small molecule inhibitors, which prevent the association of SMRT with BCL6, inhibit transcriptional repression and cause apoptosis of lymphoma cells *in vitro* and *in vivo*. In order to discover compounds, which have the potential to be developed into BCL6 inhibitors, we screened a natural product library. The ansamycin antibiotic, rifamycin SV, inhibited BCL6 transcriptional repression and NMR spectroscopy confirmed a direct interaction between rifamycin SV and BCL6. To further determine the characteristics of compounds binding to BCL6-POZ we analyzed four other members of this family and showed that rifabutin, bound most strongly. An X-ray crystal structure of the rifabutin-BCL6 complex revealed that rifabutin occupies a partly non-polar pocket making interactions with tyrosine58, asparagine21 and arginine24 of the BCL6-POZ domain. Importantly these residues are also important for the interaction of BLC6 with SMRT. This work demonstrates a unique approach to developing a structure activity relationship for a compound that will form the basis of a therapeutically useful BCL6 inhibitor.

## Introduction

**Figure 1:**
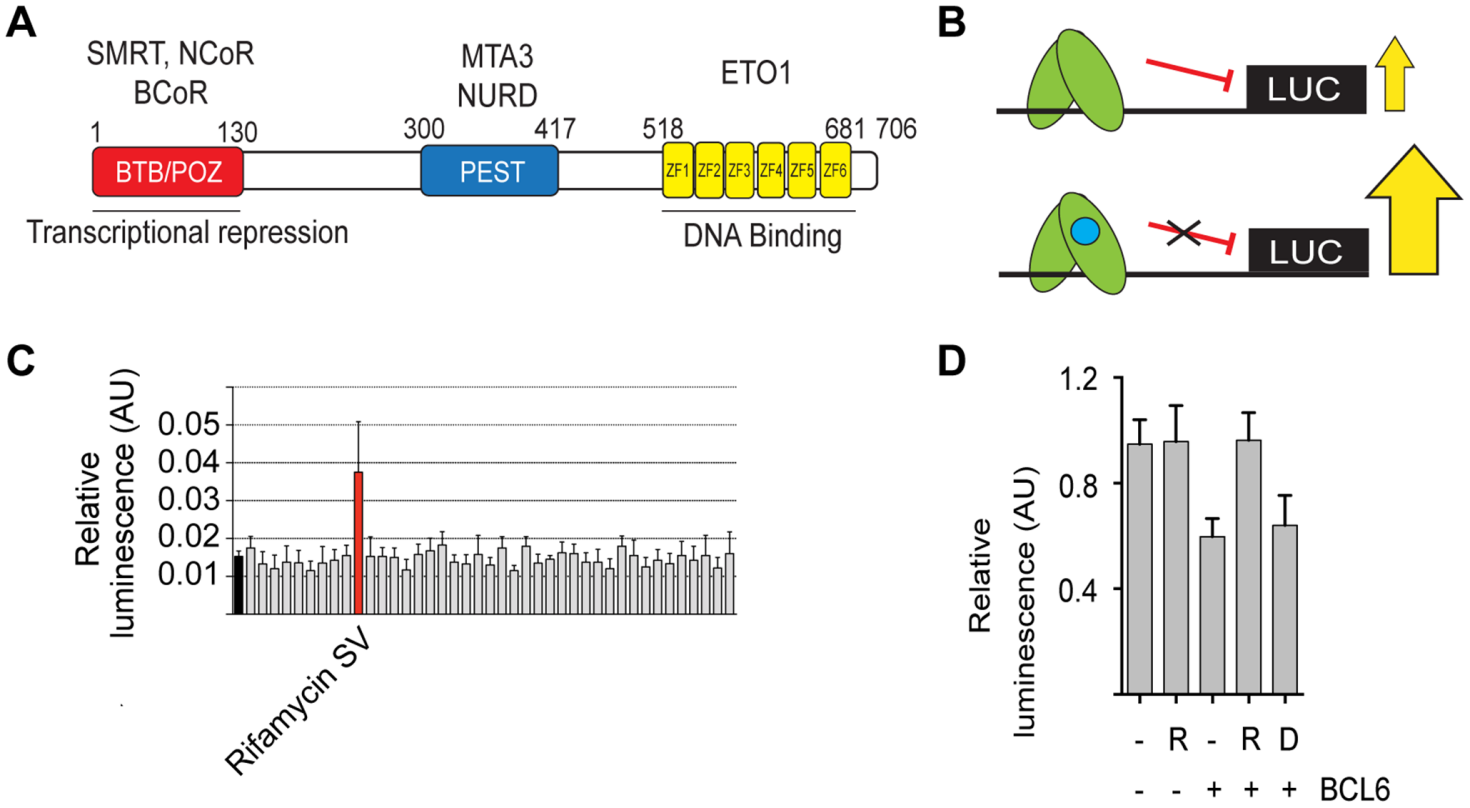
A natural product screen to identify novel inhibitors of BCL6 transcriptional repression. (A) Schematic of BCL6 showing amino-terminal POZ domain (red), carboxy terminal zinc fingers (yellow) and mid portion containing PEST domains (blue). Different proteins associate with the three portions of BCL6. NCoR, BCoR and SMRT associate with the POZ domain, MTA3 and NuRD with the mid portion and ETO1 with the zinc fingers. (B) Illustration of the screening strategy. BCL6 (green) is shown associating with its binding site cloned upstream of a luciferase reporter gene. Without any compound, or with an inactive compound i.e. one that does not bind BCL6, luciferase output is repressed but in the presence of active compound BCL6 mediated repression is prevented and output of luciferase increases. (C) Screening results for half a plate (40 compounds) from the natural product library. The black bar (furthest left) is the mean negative control i.e. transfected cells without test compound, and the black horizontal line the mean value across the entire screen. The red bar shows rifamycin SV. (D) The effect of rifamycin is due to inhibition of BCL6 transcriptional repression. HEK293T cells were co-transfected with a BCL6 expression construct and a luciferase reporter. Transcriptional repression due to BCL6 was relieved by rifamycin SV (R), but not by an agent that was ineffective in the screen (D).

BCL6 is a transcriptional repressor [1] that accomplishes its effects by binding to DNA through carboxy-terminal zinc fingers and recruitment of co-repressors to its mid-portion and amino-terminus (Figure 1A). Co-repressors NCoR (NCOR1), BCoR (BCOR) and SMRT (NCOR2), which are components of multi-protein complexes that include histone deacetylases, associate with the amino-terminal POZ domain [2]–[4]. SMRT and NCoR share an amino acid sequence (GRSIHEIPR) that is required for binding to the BCL6-POZ domain and is functionally important [5] but in contrast BCoR binding is by means of a different primary sequence (APSSWVVPG) [6]. The binding of co-repressor, SMRT, to the BCL6-POZ domain has been shown to be required for BCL6 function in B-cells, although it may be dispensable for its function in T-cells [7]. SMRT is a scaffold protein that mediates the recruitment of the HDAC3 repression complex to BCL6 and other repressive transcription factors [8].

BCL6 is expressed in normal germinal center B-cells [9] and is essential for high affinity antibody formation [10], [11]. At a cellular level its role may be to allow proliferation and inhibit differentiation to plasma cells [12]. It has been demonstrated that BCL6 promotes the proliferation of primary tonsillar B-cells [13] and prevents terminal differentiation to plasma cells in B-cell lines [12], [14].

BCL6 is involved in chromosomal translocations in ∼25% of all cases of diffuse large B-cell lymphoma (DLBCL) [15] and is, therefore, likely to have a major role in driving lymphomagenesis. This is supported by the finding that mice with constitutive B-cell expression of BCL6 develop lymphomas similar to human DLBCL [16]. Gene expression profiling has been utilized to subtype DLBCL into groups with differing clinical outcomes [17], [18]. The majority of cases with BCL6 translocations are associated with poor prognosis activated B-cell (ABC) DLBCL [19] as defined by the “cell of origin” classification [18], [20]. Other mechanisms causing constitutive expression of BCL6 have been described; mutations disrupting a negative regulatory site in the promoter region of the *BCL6* gene occur in 10 to 15% of DLBCL [21], [22] and disruption of normal post-translational regulation of BCL6 by various mechanisms have also been reported and are likely to contribute to deregulated expression [23]–[25]. Overall BCL6 is an important oncogene in DLBCL but it is also expressed from an un-rearranged locus in follicular lymphoma, Burkitt's lymphoma and nodular lymphocyte predominant Hodgkin's lymphoma. Although its role has not been investigated in detail in these diseases it is also likely to contribute to cellular proliferation and survival.

A peptide corresponding to the region of SMRT interacting with the BCL6-POZ domain has been demonstrated to be functionally active *in vitro* and *in vivo* [26], [27]. The peptide prevents normal germinal center formation in mice and when administered to BCL6 dependent cell lines or primary lymphoma cells causes apoptosis. A combination of computer assisted drug design and screens of small molecule libraries led to the identification of a compound, 79–6, that binds in the SMRT binding groove in the BCL6 POZ domain [28]. 79–6 is also functionally active in vivo and causes apoptosis of BCL6 dependent lymphoma cell lines. However, there are on-going efforts to carry out further small molecule library screens for BCL6 inhibitors.

Here we report the identification of a direct interaction between the BCL6 POZ domain and members of the ansamycin antibiotic family: rifamycin SV and rifabutin. This represents a novel non-bactericidal activity of the rifamycin family of antibiotics. Rifabutin was found to cause the largest chemical shift perturbations by NMR and a crystal structure of BCL6 in complex with rifabutin reveals new insights into the structure activity relationships required for potential therapeutic agents to disrupt the SMRT/BCL6 interaction.

## Materials and Methods

### Luciferase reporter screening assay

A BCL6 reporter construct as previously described [12] was transfected into DG75 an EBV negative Burkitt's lymphoma cell line utilising Nucleofector program O-006 (Lonza Group Ltd, Basel, Switzerland). A natural product library (TimTec, Newark, DE, USA) was purchased unsolvated and solvated to a concentration of 10 mM with DMSO. After each compound was effectively solvated, 5 μl was added to the corresponding wells in a daughter plate and diluted with 95 μl of sterile water. From the daughter plate, 3 µl of compound was pipetted into 22 μl of complete RPMI 1640 in quadruplicate into each different assay plate. The compounds were diluted into each assay plate at a concentration of 20 µM. One batch of DG-75 cells was transfected with BCL6 reporter construct with a standard amount of a construct expressing Renilla luciferase as a transfection control. The transfected cells were then incubated at 37°C for 16 to 20 hours. The assay plates were centrifuged and 50 µl of the transfected cells were pipetted into the designated wells on the assay plate. The transfected cells were incubated for 12 hours with the compounds before harvesting and determination of luciferase activity.

HEK293T cells were seeded at 2×10^4^ cells/well in 96-well plates and following 24 hours in culture were co-transfected with a BCL6 reporter vector (100 ng), Renilla luciferase control vector (100 ng) and a full-length BCL6 expression plasmid (200 ng) using polyethylenimine (PEI) (Sigma, St. Louis, MO, USA). Compound (5 µM) was added and cells were lysed, harvested and luciferase activity determined after 24 hours. Each condition was carried out in triplicate.

### Protein expression and purification

DNA encoding the POZ domain (residues 7 to 128) of human BCL6 (Figure 1A), with cysteine8 mutated to glutamine, cysteine67 mutated to arginine and cysteine84 mutated to asparagine, was cloned into a vector containing a 58-amino acid GB1 solubility enhancement tag, a 6× histidine affinity tag and a TEV cleavage site (PROTEX, University of Leicester; (http://www2.le.ac.uk/department/biochemistry/research-groups/protex)). Constructs were expressed in the *E.coli* strain Rosetta (DE3) (Novagen, Merck Chemicals Ltd., Beeston, UK) (Figures S1A and S1B). For preparation of ^15^N-labelled samples bacteria were cultured 2M9 minimal media containing 1 g of ^15^N-ammonium chloride per liter. For crystallisation and fluorescence polarisation *E. coli* were cultured in 2xYT medium. Bacteria were cultured at 37°C BCL6-POZ was purified using Ni-NTA resin and subsequent buffer exchange into 50 mM sodium phosphate pH 6, 300 mM NaCl, 5 mM DTT. Following TEV cleavage overnight at 4°C the sample was further purified by gel filtration using a Superdex S200 column (GE Healthcare, Amersham, UK). Protein concentrations were measured using Bio-Rad Protein Assay (Bio-Rad, Hercules, CA, USA).

### Peptide Synthesis and Fluorescence Polarization

Fmoc-protected amino acids were purchased from Novabiochem (Merck Chemicals Ltd, Nottingham, UK) or PolyPeptide Group (Strasbourg, France) (Fmoc-homophenylalanine, Fmoc-Styrylalanine, Fmoc-1-naphthylalanine & Fmoc-2-naphthylalanine) and were used as received. Peptides were synthesized on a CEM Liberty 1 automated microwave-assisted solid-phase peptide synthesizer (CEM Corporation, Buckingham, UK) using a 30 mL Teflon reactor vessel on 0.05 mmol scale using Fmoc-Arg(Pbf)-Wang resin (100–200 mesh) (substitution: 0.63 mmol/g). Peptide solutions were made in PBS containing 1 mM tris-(2-carboxyethylphosphine) and then coupled via the amino-terminal cysteine to the thiol-reactive BODIPY TMR dye (Invitrogen, Paisley, UK) in accordance with manufactures instructions. Unreacted dye was removed by gel filtration using a PD-10 column (GE Healthcare). Fluorescence polarization experiments were performed in a black 96 well assay plate (Corning, Amsterdam, The Netherlands). Titrations were performed using a fixed concentration of SMRT peptide, with increasing concentration of the BCL6-POZ domain protein, in a final volume of 100 μl of assay buffer (PBS, 0.05% (v/v) Triton X-100, 0.1 mg/mL BSA). The plate was mixed by shaking for 1 min and measurements were then taken using a Victor X5 plate reader (Perkin Elmer, Waltham, MA, USA) at room temperature with an excitation wavelength of 531 nm and an emission wavelength of 595 nm. Experiments were performed in triplicate and data were analysed using GraphPad Prism (version 6.0, GraphPad Software, Inc., San Diego, CA, USA). K_d_ values were calculated by nonlinear curve fitting using a one-site binding (hyperbola).

### NMR spectroscopy

All NMR experiments were performed at 303 K using Bruker AVANCE DRX 600 or AVANCE AVII 800 spectrometers both equipped with CryoProbes. Titrations were carried out using 280 μM BCL6-POZ in 50 mM sodium phosphate pH 6, 300 mM NaCl, 5 mM DTT, 5% v/v D_2_O. Compounds were resuspended in deuterated DMSO (DMSO-d6). 2D ^1^H^15^N heteronuclear single-quantum correlation (HSQC) spectra were acquired with transverse relaxation optimization (TROSY) [29] using 32 scans and 92 increments. ^1^H^15^N HSQC spectra were collected on BCL6-POZ alone and then with increasing amount of compound. Data were analyzed using CCPN Analysis [30].

### Crystallization and X-ray structure determination

Crystals of the BCL6-POZ domain were obtained using the sitting drop vapor diffusion method at room temperature (Figure S1) BCL6-POZ was concentrated to 3.8 mg/ml and crystallised in the presence of rifabutin at a ratio of 1∶8. In detail 1 μl of BCL6-POZ in 50 mM sodium phosphate pH 6, 300 mM NaCl, 5 mM DTT (in the presence or absence of rifabutin) was mixed with 1 μl reservoir solution (20% PEG 6000, 100 mM sodium citrate, pH 5). Crystals grew in the space group P1 21 1. Data were collected to 2.3 Å on the microfocus beam line I24 at the Diamond Light Source, Didcot, Oxfordshire. Data were processed and integrated using XDS, iMosflm, Pointless and Aimless [31], [32]. The structure was solved using molecular replacement using Phaser [33] and the BCL6-POZ domain from the BCL6/SMRT structure (1R2B, [34]). Model fitting and refinement were performed using Coot and Refmac [35], [36]. Statistics of the refinement are presented in Table 1. The R_free_ remained higher than expected probably due to the small size of the crystals and slightly streaky nature of the diffraction.

**Table 1:** Data collection and refinement statistics (Molecular replacement).

BCL6/Rifabutin	
**Data Collection**	
Space Group	P 1 21 1
Cell dimensions	
* a,b,c* (Å)	35.17, 54.83, 58.16
* α,β,γ* (°)	90, 95.21, 90
Resolution (Å)	39.82–2.3 (2.38–2.3)
R_merge_	10.8 (51.8)
I/σI	9.8 (4.1)
Completeness (%)	97.13 (97)
Redundancy	3.0 (2.9)
**Refinement**	
Resolution (Å)	2.3
No. reflections	9168
R_work_/R_free_	20.2/26.9
No. Atoms	2053
Protein	1969
Ligand/ion	61
Water	23
B-factors	
Protein	27.9
Rifabutin	48
Water	24.6
R.M.S. deviations	
Bond lengths (Å)	0.013
Bond angles (°)	1.885

*Highest resolution shell is shown in parenthesis.

### Accession numbers

Coordinates and structure factors for the BCL6-POZ domain (residues 7–128) – Rifabutin complex have been deposited in the Protein Data Bank (ID code 4CP3).

## Results

### Natural Product Screen for inhibitors of the BCL6-SMRT interaction

Natural products form the basis for many drugs in clinical use. Despite their chemical complexity, they have other properties such as cell permeability and relatively high bioavailability that make them attractive starting materials for screens in drug discovery projects. We screened a commercial natural product library consisting of 480 compounds for ability to prevent BCL6 induced transcriptional repression in the Burkitt's lymphoma cell line DG75 (Figure 1B). Nine compounds modified transcriptional activity of a reporter construct bearing BCL6 binding sites in DG75 (3 compounds repressing and 6 compounds enhancing luciferase activity) (Figure 1C and Figures S2 and S3). However, the BCL6 DNA binding sequence shares sequence similarities to that of the STAT family of transcription factors [10] and our results might reflect inhibition of transcription factors other than BCL6. Therefore, to show directly that BCL6 transcription was inhibited we co-transfected a BCL6 expression construct and a luciferase reporter into HEK293T cells that do not express endogenous BCL6. Luciferase expression was repressed by BCL6 and this was relieved by the addition of rifamycin SV (labelled R) (Figure 1D), which was detected in the initial library screen (Figure 1C), but not by the other eight compounds.

### Rifamycin SV directly interacts with BCL6-POZ

**Figure 2:**
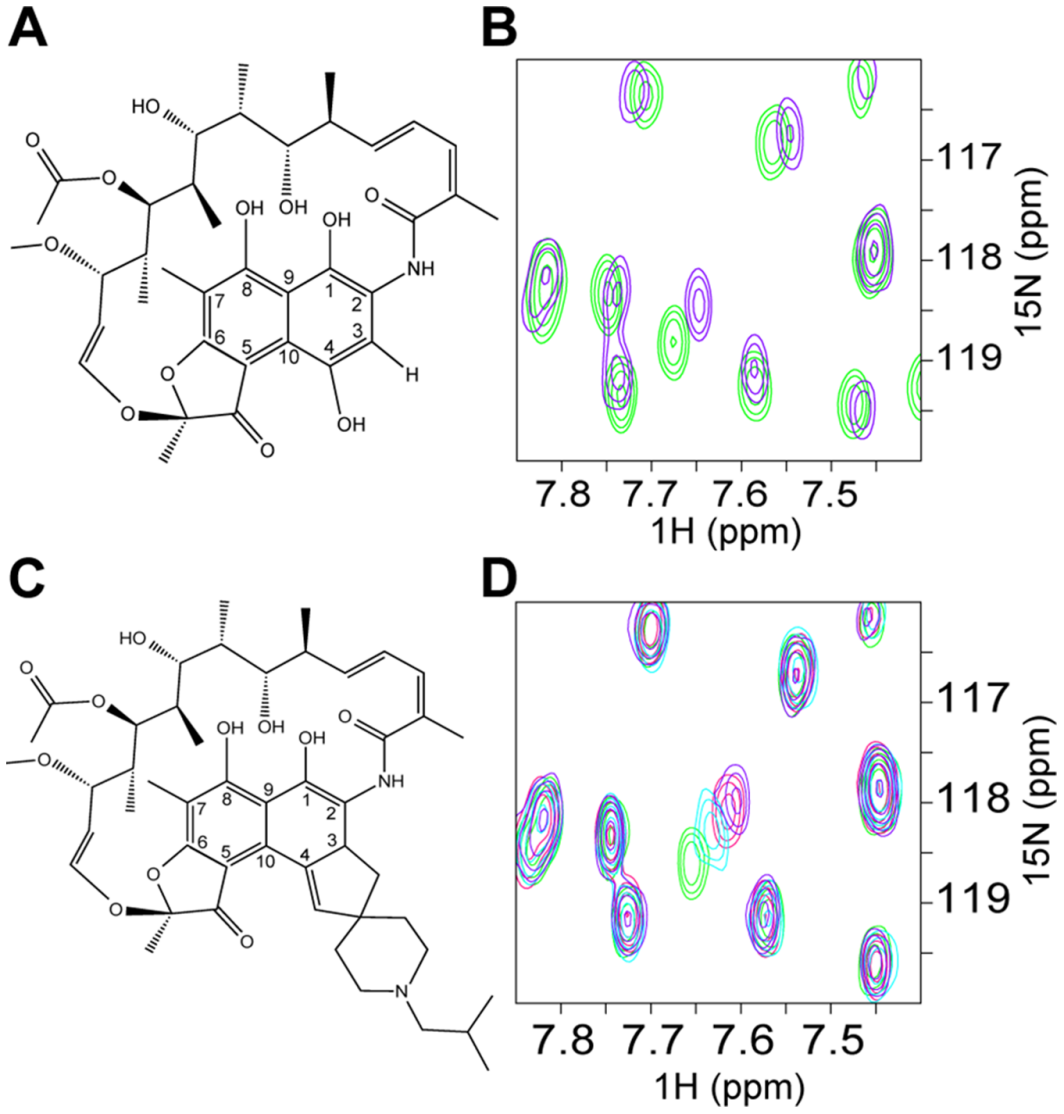
Rifamycin SV and its derivative, rifabutin, bind directly to the BCL6-POZ domain. Schematic diagram to compare the structures of (A) rifamycin SV and (C) rifabutin. These two compounds differ with respect to the side chains on C-3 and C-4. (B) and (D) TROSY ^1^H,^15^N HSQC spectra of 280 μM BCL6-POZ domain. (B) Chemical shift changes due to rifamycin SV. An overlay of the spectra of BCL6-POZ domain alone (green) and in the presence of a 16∶1 molar ratio of rifamycin (purple). (D) Chemical shift changes due to rifabutin with an overlay of the spectra of BCL6-POZ domain alone (green) and in the presence of 4∶1 (light blue), 8∶1 (red) and 16∶1 (purple) molar ratios of rifabutin.

In order to determine whether a direct interaction with BCL6 in solution was responsible for the observed effects on transcription, we utilized an NMR chemical shift perturbation assay. Work by others has demonstrated that the important interactions of BCL6 with co-repressors occur through the amino-terminal POZ domain, which also mediates homodimerisation [37]. The TROSY ^1^H^15^N TROSY-HSQC NMR spectrum of the BCL6-POZ homodimer showed good dispersion and uniform line widths indicative of a stable well-folded protein (Figure S4). Addition of rifamycin SV (Figure 2A), resulted in a subset of peaks shifting in a concentration dependent manner (Figure 2B) supporting the hypothesis that rifamycin interacts directly with the BCL6-POZ domain. However, even at large excesses of rifamycin SV the chemical shift perturbations were relatively small and chemical shift changes continued to be detectable at high concentrations demonstrating that binding had still not reached saturation.

### Screening of rifamycin derivatives

Rifamycin SV belongs to a family of ansamycin antibiotics, which have related structures, comprising a napthoquinone ring bridged by an aliphatic chain (Figure 2A and 2C). In order to determine whether other members of the family also bound to the BCL6-POZ domain we analysed the commercially available compounds rifabutin, rifapentine, rifampicin and rifaximin as well as 3-formyl rifamycin, for interaction with the BCL6-POZ domain.

These derivatives all caused spectral changes of varying magnitude with the greatest shifts caused by rifabutin (Figure 2C and 2D). The estimated order of binding from weakest to strongest was: rifaximin, rifapentine, 3-formyl rifamycin, rifampicin, rifamycin SV and rifabutin. Comparison of the BCL6-POZ domain spectra on addition of rifamycin SV and rifabutin (Figures 2B and 2D) showed small differences in the observed shifts of some peaks suggesting minor differences in binding in solution. By plotting the chemical shift change (δΔ) of the most shifted peak, as a function of rifabutin concentration it was possible to estimate the Kd of the interaction as being in the order of ∼1 mM.

### Structure of the BCL6-POZ- Rifabutin complex

**Figure 3:**
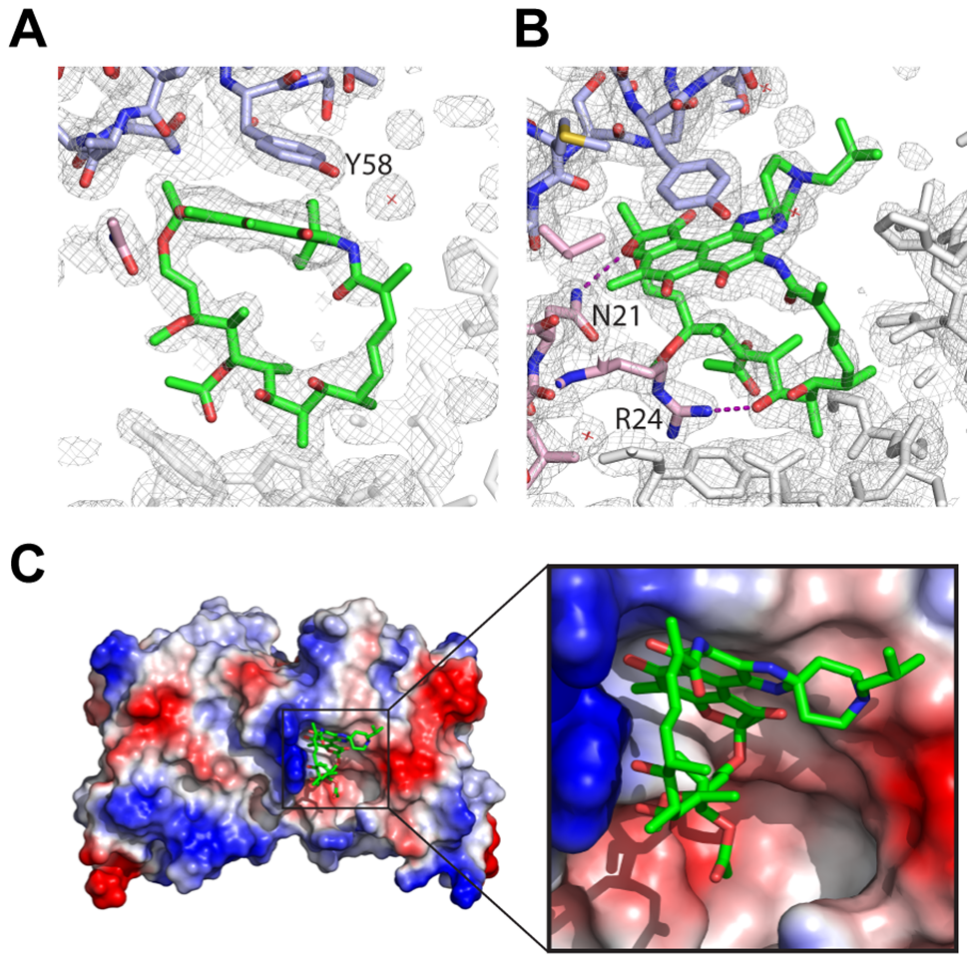
Crystal structure of rifabutin and BCL6-POZ domain. Electron density corresponding to rifabutin, following refinement, in the context of surrounding electron density demonstrating the proximity of (A) tyrosine58 from one monomer of the POZ dimer and (B) asparagine21 and arginine24 from the other monomer. (C) Surface representation of BCL6-POZ with basic residues (including asparagine21 and arginine24) in blue and acidic residues in red. The napthoquinone rings of rifabutin are in proximity to tyrosine58 whilst the aliphatic bridge is adjacent to the basic surface.

To explore further the atomic details of the interaction we crystallized the BCL6-POZ domain in complex with rifabutin. Complex crystals were readily obtained and easily identified due to the purple colour of rifabutin (Figure S1C). Molecular replacement with the BCL6-POZ domain (1R2B, [34]) as search molecule produced a clear electron density map with extra density readily identifiable for rifabutin (Figure 3, Table 1). Despite the BCL6-POZ domain being a symmetrical dimer [2], [3] only one molecule of rifabutin is present per dimer. The rifabutin is located at the dimer interface and binds to the surface that overlaps the surface bound by the SMRT and NCoR peptides [2], [34]. The napthoquinone ring of rifabutin occupies the pocket, which is occupied by the residues histidine1426 of SMRT, histidine1352 of NCoR, or tryptophan509 of BCOR, in the three POZ domain co-repressor structures [6]. The most important interaction appears to be a π-stacking interaction with the aromatic ring of tyrosine58 of the BCL6-POZ domain. The aliphatic “handle” or macrocycle of rifabutin makes electrostatic interactions with asparagine21 and arginine24 (Figure 3).

**Figure 4:**
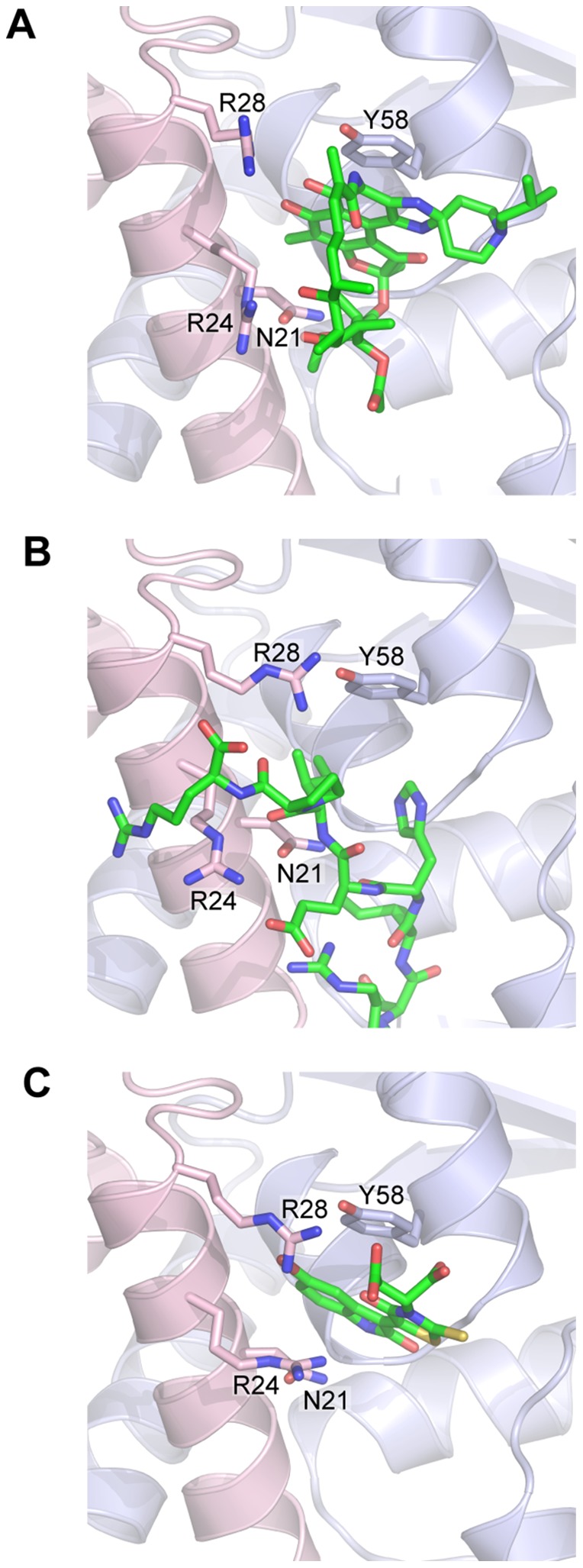
BCL6-POZ domain in complex with rifabutin, 79–6 and the SMRT peptide. A section of the BCL6-POZ domain structure is shown binding; (A) rifabutin, (B) the SMRT peptide and (C) 79–6.

A small molecule, 79–6, has been described previously and has been observed to bind in the same pocket at the BCL6:SMRT interface [28]. Comparison of the binding of SMRT peptide, 79–6 and rifabutin demonstrates remarkable similarities (Figure 4). Specifically apolar interactions with tyrosine58 and electrostatic interactions with asparagine21 and arginine24 are involved in the binding of all three molecules.

### SMRT peptide containing artificial amino acids to explore binding to BCL6-POZ domain

**Figure 5:**
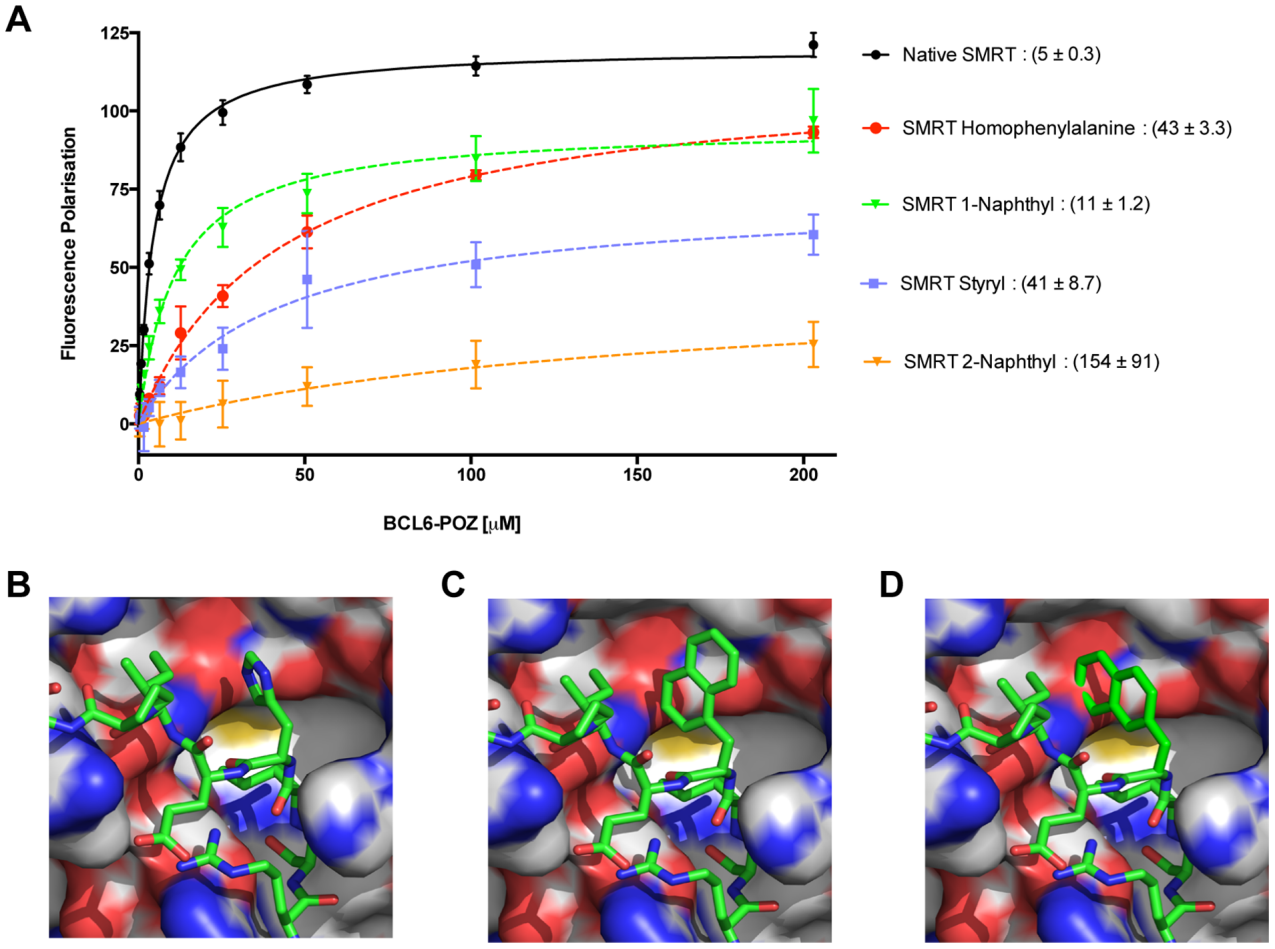
Binding requirements of the SMRT peptide explored utilising artificial amino acids to replace histidine. (A) Binding curves obtained by fluorescence polarisation for wild-type peptide and 1-napthyl, 2-napthyl, styryl and homophenylalanine substitutions of histidine1426 of SMRT. The K_d_ in µM (mean±SEM) is presented to the right of the compound name. (B) Structure of wild-type SMRT peptide bound to the BCL6-POZ domain. Molecular modelling of (C) 1-naphthyl SMRT peptide and (D) 2-naphthyl SMRT peptide.

In order to explore the importance of histidine1426 of SMRT or histidine1352 of NCoR for binding to the pocket in the BCL6-POZ domain, which is also occupied by rifabutin and 79–6 we synthesised SMRT/NCoR peptides with artificial amino acids replacing the histidine (Figure S5). By fluorescence polarisation the K_d_ of binding of the BCL6-POZ domain to labelled wild-type SMRT peptide was determined to be 5 µM. All the artificial amino acids that were employed in the study showed reduced binding, as compared to wild-type peptide, but there were considerable differences in affinity. Whilst the peptide bearing a 1-naphthyl residue had a binding affinity of 11 µM the 2-naphthyl peptide had a much lower affinity of 154 µM (Figure 5A). Homophenylalanine and styryl derivatives had intermediate affinities. Modelling of these artificial amino acids, such that they have the same orientation as tryptophan509 of BCOR and histidine1426 of SMRT [6], suggested explanations for this data (Figure 5B, 5C and 5D). Whilst 1-naphthyl is oriented within the pocket, 2-naphthyl clashes with the BCL6-POZ domain, which is likely to prevent significant binding. Homophenylalanine and styryl side chains were employed to place aromatic rings, potentially capable of interacting with tyrosine58 closer to the BCL6-POZ domain than is histidine1426 in the wild-type structure. Binding affinity was again reduced demonstrating the stringent requirements for compounds that are to be lead molecules as BCL6 inhibitors.

## Discussion

There is interest in developing BCL6 inhibitors because this transcription factor is required for proliferation and survival of several types of non-Hodgkin's lymphoma and nodular lymphocyte predominant Hodgkin's lymphoma and proof of principle studies have demonstrated the efficacy of inhibiting BCL6 in diffuse large B-cell lymphoma [27], [28]. The rifamycins and especially rifabutin are attractive starting materials for producing a clinically useful BCL6 inhibitor because of their high lipid solubility, extensive tissue penetrance and long half-life [38].

We have demonstrated that rifamycin SV and rifabutin, members of the ansamycin antibiotic family, that have clinical uses in the prevention or treatment of bacterial infections due to binding and inhibition of bacterial DNA-dependent RNA polymerase, are able to bind the BCL6-POZ domain.

This result is functionally significant as demonstrated by inhibition of BCL6 transcriptional repression by rifamycin SV. The luciferase reporter assay we employed produced 9 “hits” of which only rifamycin SV bound to BCL6. Three of the compounds appeared to enhance transcriptional repression i.e. reduced luciferase production, and for one of these compounds (Figure S2A) the explanation may be that it is a known inhibitor of firefly luciferase (Figure S3B) [39].

Rifamycin SV is derived from rifamycin B (the natural fermentation product of *Streptomyces mediterranei*) by removal of the glycolic group bound to C-4. The rifamycins are potent inhibitors of bacterial DNA dependent RNA polymerase [40] and are utilised in the treatment of tuberculosis. Another property of these compounds, their high lipid solubility, may help to penetrate the bacterial wall. Structurally, the rifamycins consist of a naphthoquinonic chromophore, which is spanned by an aliphatic bridge between the nitrogen on C-2 and the oxygen on C-12 of the napthoquinone moiety (Figure 2A and 2C). The members of the family differ primarily in the side chains on C-3 and C-4. We demonstrate that rifamycins bind with different strengths to the BCL6 POZ domain. These agents largely differ in the side chain attachments to C-3 and C-4, which do not appear to make significant interactions with the protein in our crystal structure. One possibility is that the side-chains alter the rigidity of the molecule to alter the fit in the lateral groove of the POZ domain dimer. Supporting the view that the C-3/C-4 side chains are important in modulating function, others have shown that rifamycin SV can inhibit amyloid fibril formation through disruption of interactions between fibril aromatic rings that are required for elongation whereas rifaximin does not have this effect [41].

SMRT and NCoR occupy a binding pocket that is present in the apo i.e. unliganded, form of BCL6. The co-repressor, BCOR, associates with BCL6 through interactions with the lateral groove as do SMRT and NCoR. Whilst SMRT and NCoR bind to the BCL6-POZ domain through identical sequences, GRSIHEIPR (residues 1422 to 1430 of SMRT and residues 1348 to 1356 of NCoR), there is no similarity to the BCL6 binding sequence of BCoR, APSSWVVPG. However, binding of peptides derived from SMRT and BCoR have been investigated in detail [6]. BCOR tryptophan509 and SMRT histidine1426 make similar contacts on the BCL6-POZ domain, namely with residues methionine51, cysteine53, serine54, glycine55, asparagine21, arginine24 and arginine28 [6]. The small molecule, 79–6, utilizes a subset of the interactions employed by the SMRT peptide for binding to the BCL6 POZ domain. Our X-ray crystallographic studies show that rifabutin occupies the pocket utilised by histidine in the SMRT and NCoR sequences and tryptophan in BCoR. The binding of rifabutin to the BCL6 POZ domain also appears very similar to that of the small molecule, 79–6, [28]. Collectively it seems likely that rifamycin SV and rifabutin can occupy a druggable pocket in the BCL6-POZ domain.

Macrocyclic compounds, of which rifamycin SV and rifabutin are examples, may have utility in perturbing protein-protein interactions and are also highly membrane permeable. We suggest that the rifamycins will be interesting compounds from which to develop BCL6 inhibitors.

## Supporting Information

Figure S1 **Protein purification and crystallisation of BCL6.** (A) Coomassie stained polyacrylamide gel showing production of BCL6-POZ domain with a GB1 tag and TEV cleavage of the tag. (B) BCL6-POZ domain purified away from the GB1 tag by size exclusion gel filtration. Coomassie stained polyacrylamide gel showing fractions collected. (C) BCL6-POZ crystal mounted in a loop at the Diamond Synchrotron beamline I24 (Red box 25.9 μm^2^). The crystals are faintly lilac having taken up the coloured rifabutin.
(TIF)

Figure S2 **Natural product library screening.** Results for all 480 compounds are presented. Column to the left in orange is the mean negative control i.e. transfected cells without test compound, and the orange horizontal line the mean value across the entire screen. Compounds considered “its”are represented as red columns and comprise 6 that relieve BCL6 transcriptional repression and 3, which appear to enhance repression.
(TIF)

Figure S3 **Structures of the 9 compounds that alter BCL6 transcriptional repression.** The most widely used compound name is presented apart from one compound (A), which has no common name and for which the IUPAC nomenclature is stated. The chemical identifier (CID) from PubChem is also presented. (A to C) Three compounds that reduce luciferase activity. (D to I) Compounds that enhance luciferase activity, including (I) Rifamycin SV.
(TIF)

Figure S4 **TROSY ^1^H, ^15^N HSQC NMR spectrum of the BCL6-POZ domain.**
(TIF)

Figure S5 **Sequences of SMRT peptides.** Sequences of SMRT peptides utilised in fluorescence polarisation experiments together with the structures of the artificial amino acids at the position of histidine1426 in the wild-type peptide. (A) wild-type SMRT, (B) 1-naphthyl-SMRT, (C) 2-naphthyl-SMRT, (D) homophenylalanine-SMRT and (E) styryl-SMRT.
(TIF)
