# Supplementary figures and images for: The Ansamycin Antibiotic, Rifamycin SV, Inhibits BCL6 Transcriptional Repression and Forms a Complex with the BCL6-BTB/POZ Domain
Source: PLoS One. 2014 Mar 4;9(3):e90889. doi: 10.1371/journal.pone.0090889 (PMC3942486; doi:10.1371/journal.pone.0090889)

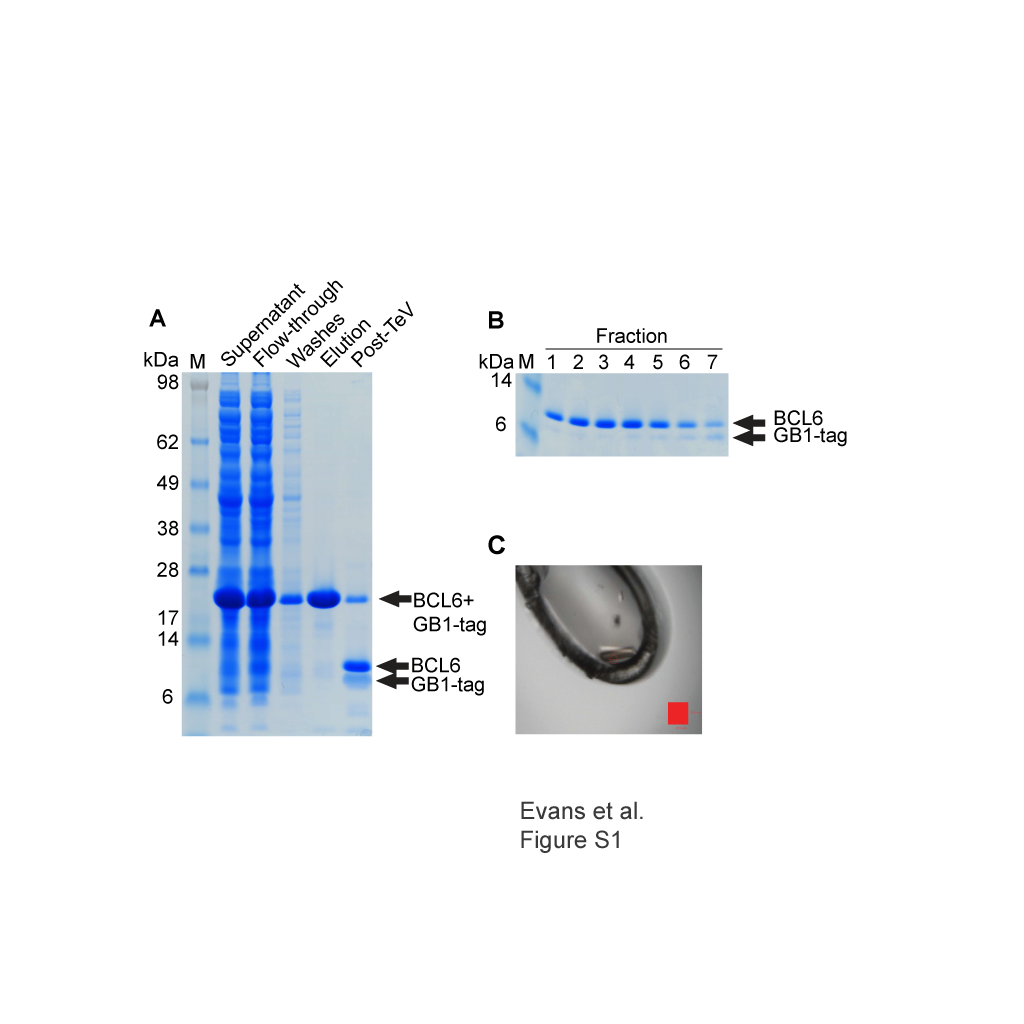

Supplement: Figure S1 — Protein purification and crystallisation of BCL6. (A) Coomassie stained polyacrylamide gel showing production of BCL6-POZ domain with a GB1 tag and TEV cleavage of the tag. (B) BCL6-POZ domain purified away from the GB1 tag by size exclusion gel filtration. Coomassie stained polyacrylamide gel showing fractions collected. (C) BCL6-POZ crystal mounted in a loop at the Diamond Synchrotron beamline I24 (Red box 25.9 μm2). The crystals are faintly lilac having taken up the coloured rifabutin. (TIF) [file pone.0090889.s001.tif]

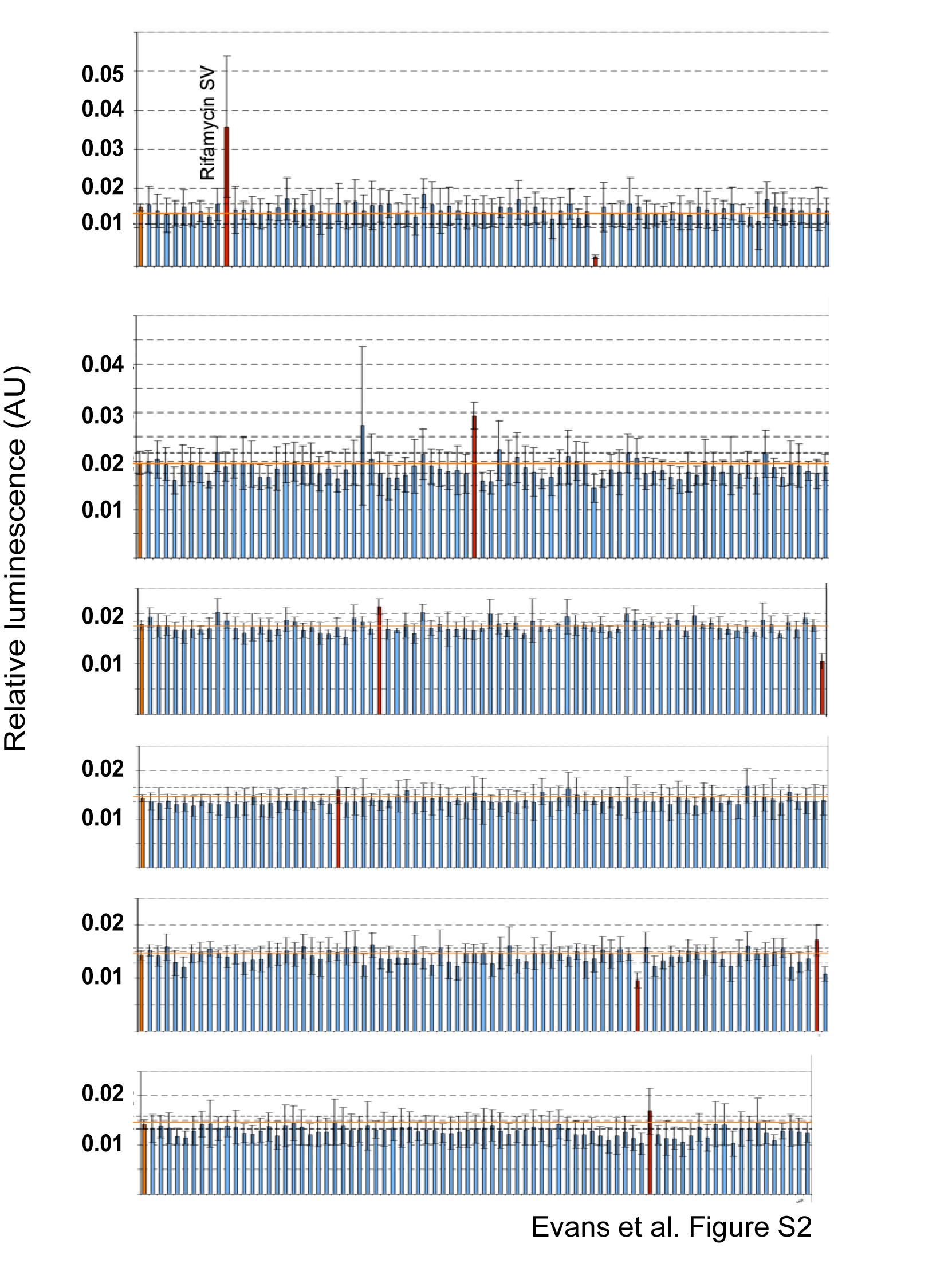

Supplement: Figure S2 — Natural product library screening. Results for all 480 compounds are presented. Column to the left in orange is the mean negative control i.e. transfected cells without test compound, and the orange horizontal line the mean value across the entire screen. Compounds considered “its”are represented as red columns and comprise 6 that relieve BCL6 transcriptional repression and 3, which appear to enhance repression. (TIF) [file pone.0090889.s002.tif]

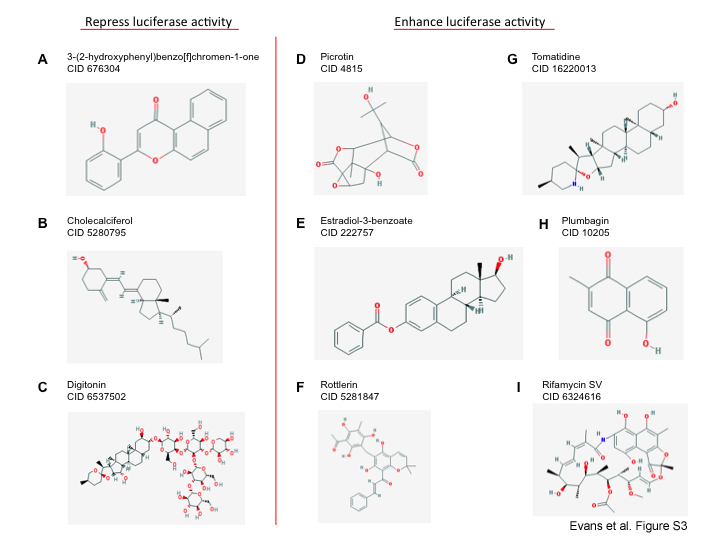

Supplement: Figure S3 — Structures of the 9 compounds that alter BCL6 transcriptional repression. The most widely used compound name is presented apart from one compound (A), which has no common name and for which the IUPAC nomenclature is stated. The chemical identifier (CID) from PubChem is also presented. (A to C) Three compounds that reduce luciferase activity. (D to I) Compounds that enhance luciferase activity, including (I) Rifamycin SV. (TIF) [file pone.0090889.s003.tif]

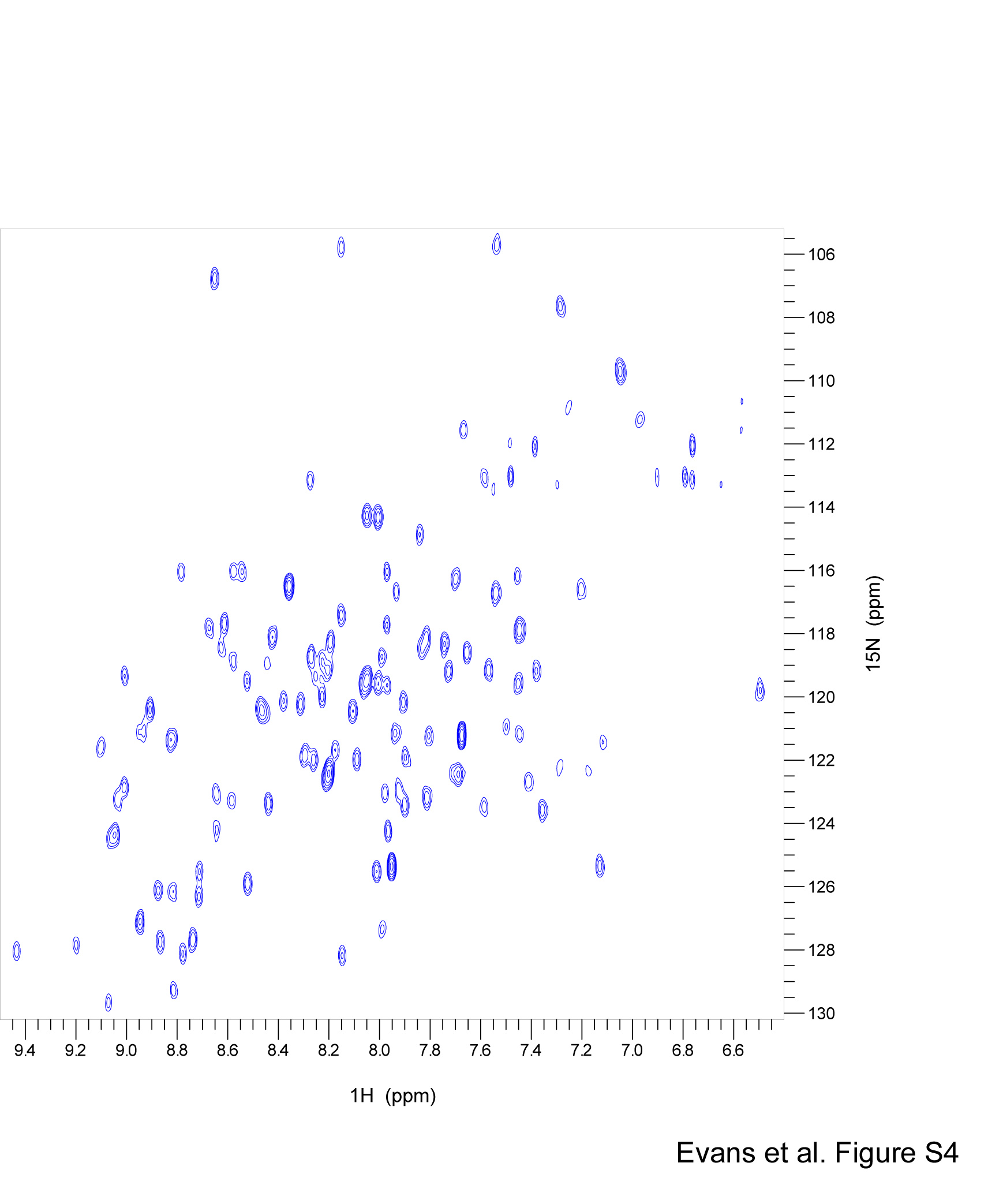

Supplement: Figure S4 — TROSY 1H, 15N HSQC NMR spectrum of the BCL6-POZ domain. (TIF) [file pone.0090889.s004.tif]

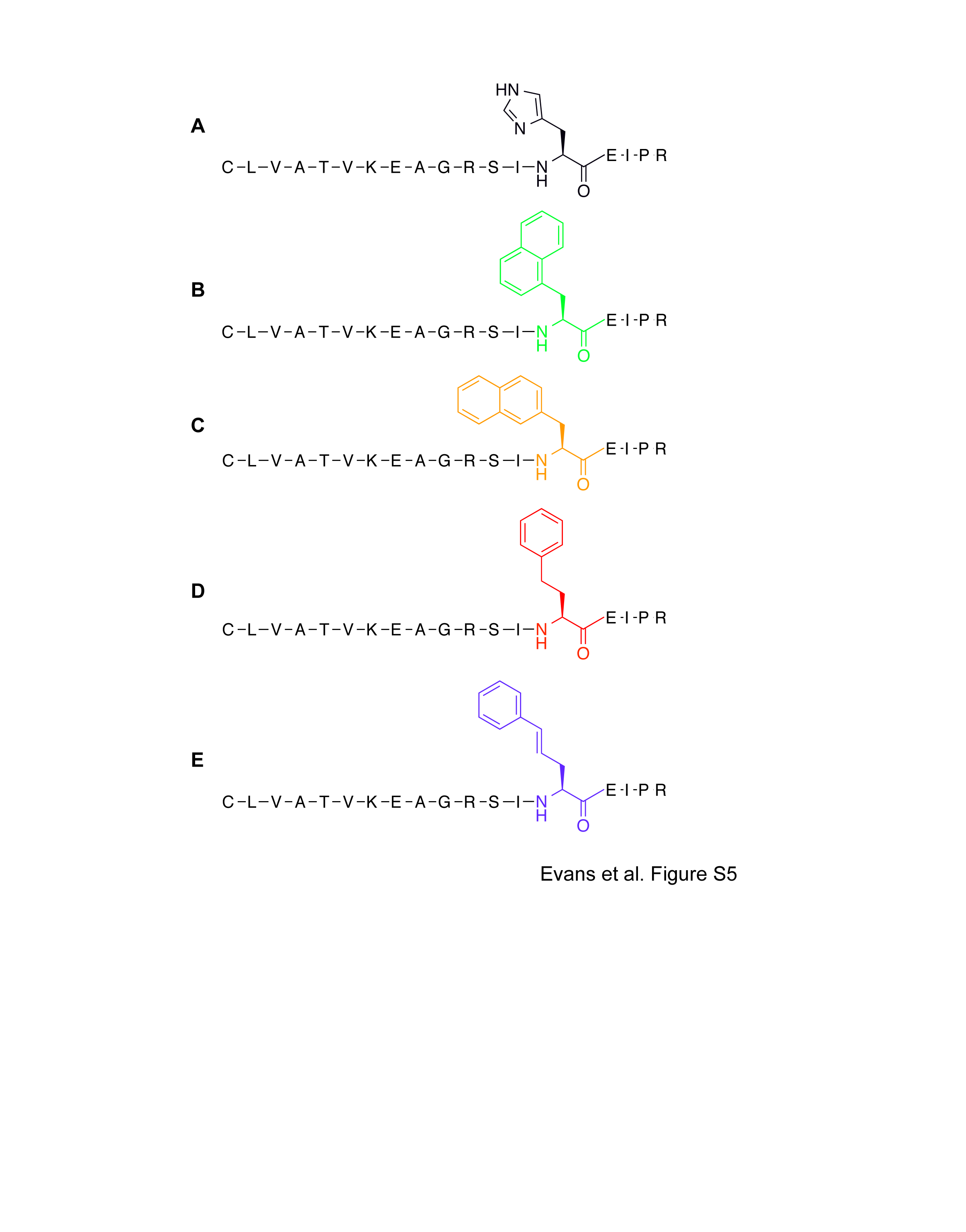

Supplement: Figure S5 — Sequences of SMRT peptides. Sequences of SMRT peptides utilised in fluorescence polarisation experiments together with the structures of the artificial amino acids at the position of histidine1426 in the wild-type peptide. (A) wild-type SMRT, (B) 1-naphthyl-SMRT, (C) 2-naphthyl-SMRT, (D) homophenylalanine-SMRT and (E) styryl-SMRT. (TIF) [file pone.0090889.s005.tif]
